# The Rise and Fall of Well-Controlled Blood Pressure: Labile Hypertension Following Repair of a Ruptured Abdominal Aortic Aneurysm

**DOI:** 10.7759/cureus.56880

**Published:** 2024-03-25

**Authors:** Grace Ansah, Madeline Conaway, Shana Childress, Kristin Slater, Paul Vellozo

**Affiliations:** 1 Internal Medicine, New York Institute of Technology College of Osteopathic Medicine, Jonesboro, USA; 2 Internal Medicine, Lincoln Memorial University, DeBusk College of Osteopathic Medicine, Harrogate, USA; 3 Internal Medicine, Lawrence Memorial Hospital Family Medical Center, Walnut Ridge, USA

**Keywords:** endovascular aortic repair, hypertension, secondary hypertension, vascular surgery, cardiology, ruptured aaa, male smoker, abdominal aortic aneurysm, labile hypertension, resistant hypertension

## Abstract

Hypertension is a common pathology with several etiologies. If left uncontrolled, severe and even fatal complications can develop, including heart disease, vascular damage, and stroke. Primary hypertension is most commonly seen without an underlying etiology; however, several contributing factors can lead to the development of hypertension. There have been limited cases reporting the effects of an abdominal aortic dissection treated with endovascular aortic repair (EVAR) on the development of labile hypertension. We report a case of uncontrolled, labile hypertension following an EVAR of an abdominal aortic aneurysm in a patient without prior medical history of hypertension.

## Introduction

Hypertension is among the most common chronic conditions in adults and is considered a leading risk factor for disability and premature deaths worldwide [1]. Hypertension can be divided into several categories, including primary (essential), secondary, and labile [2]. The majority of hypertensive cases are considered "primary," or associated with no underlying etiology or medical condition [3]. Increasing age, high dietary sodium intake, obesity, and family history are associated with the development of primary hypertension [3]. In contrast, secondary hypertension is caused by an underlying medical condition, such as renal artery stenosis, pheochromocytoma, or Cushing’s syndrome [3]. Resistant hypertension occurs when blood pressure (BP) is uncontrolled despite a regimen of three or more drugs, including a diuretic, and raises suspicion for secondary hypertension [1]. Labile hypertension is not well defined or standardized but is thought to be a rapid, short-term increase in BP to a level greater than 140/90 [4]. Labile hypertension may be related to emotional stress, pseudopheochromocytoma, or supine hypertension [4].

An abdominal aortic aneurysm (AAA) is a progressive, irreversible dilation of the aorta that is 50% greater than its normal diameter. The most important risk factors for the development of an AAA are smoking, male gender, Caucasian race, and increasing age. A large aneurysm diameter and continued tobacco use are associated with an increased risk of rupture of an AAA [5]. Here we present a case of a 71-year-old Caucasian male with a history of tobacco use who developed uncontrolled labile hypertension following repair of a ruptured AAA.

## Case presentation

**Figure 1:**
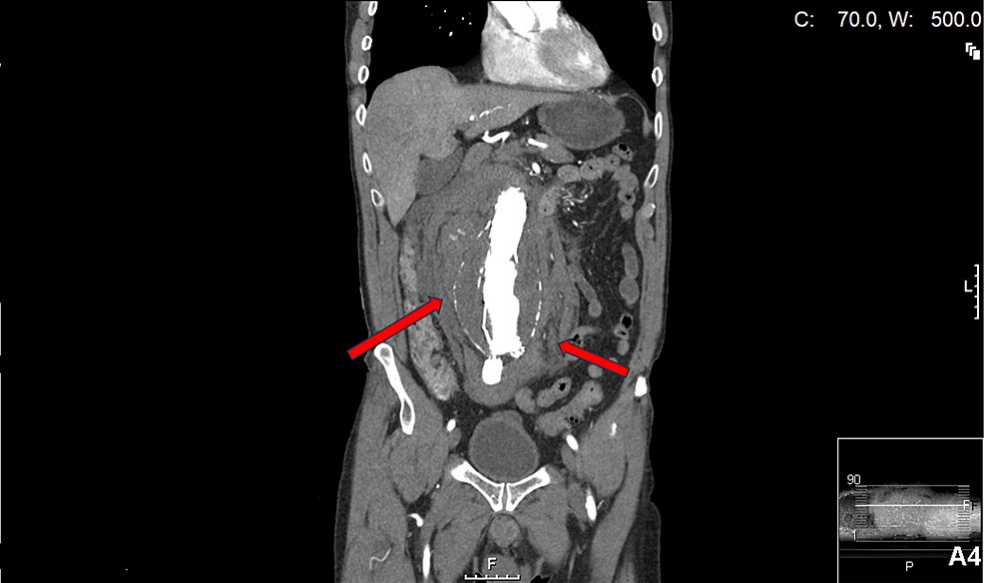
CT abdomen and pelvis coronal view showing AAA with hypo- and hyperdense-blood products spilling into the abdomen and pelvis CT: computed tomography; AAA: abdominal aortic aneurysm

**Figure 2:**
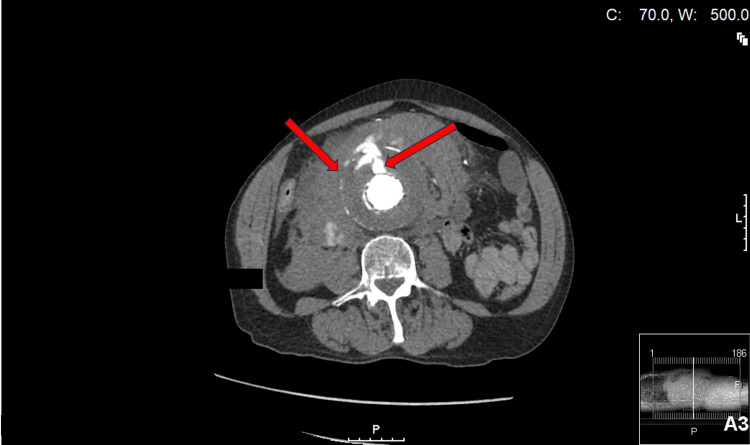
CT abdomen transverse view of AAA showing intraperitoneal blood products (left arrow) and anterior endoleak (right arrow) CT: computed tomography; AAA: abdominal aortic aneurysm

A 71-year-old Caucasian male with a prior history of AAA endovascular aortic repair (EVAR) 10 years ago, hyperlipidemia, and tobacco use with a 60-pack per year history presented to the clinic with complaints of abdominal pain. A computed tomography angiography (CTA) of the abdomen/pelvis with contrast showed interval enlargement of the native sac measuring up to 8.0 cm, anterior and posterior type III endoleaks, as well as rupture of the native AAA with a large amount of hypo- and hyperdense-blood products spilling into the abdomen and pelvis, indicative of a surgical emergency (Figures 1-2). The patient was emergently transferred to undergo an EVAR. The patient denied any ongoing tobacco use, indicating smoking cessation 25 years prior to this encounter. Before the procedure, the patient’s BP was controlled without the need for antihypertensive medications. Post-operatively, there were no complications, and the patient was placed on carvedilol 12.5 mg twice daily for elevated BP with a systolic BP (SBP) of up to 160-170. Four months later, the patient developed labile hypertension. His antihypertensive regimen was adjusted, and he was started on lisinopril 20 mg with chlorthalidone 25 mg daily. Prior to follow-up, the patient reportedly took minoxidil, required 1/2 a tablet of clonidine, and doubled his dose of carvedilol due to his increasing BP levels maximizing at 175/100. After taking the reported medications, his in-office BP dropped to 84/54 with associated lightheadedness, weakness, and pallor, thus requiring hospitalization with treatment including intravenous fluids and close monitoring.

**Figure 3:**
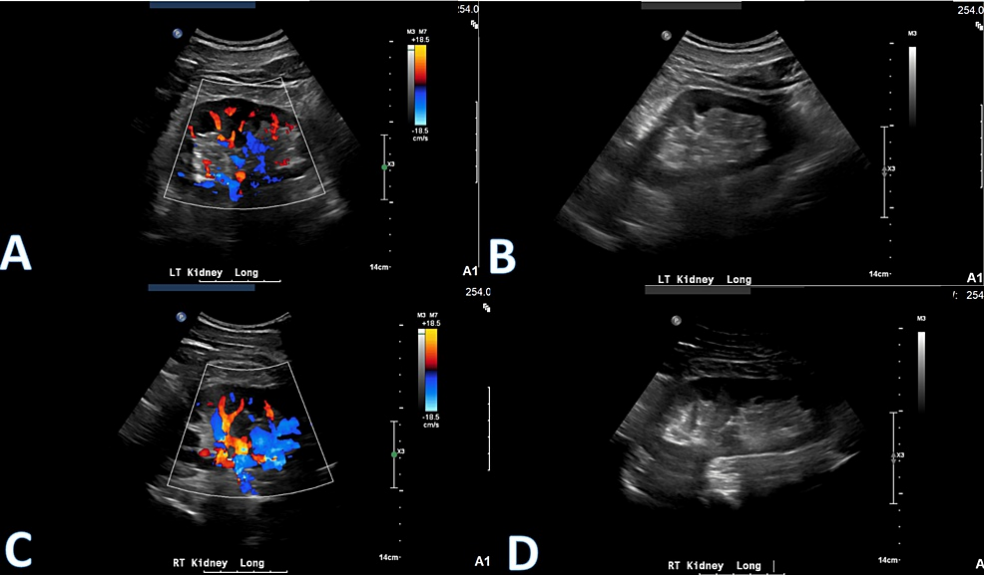
Bilateral renal artery Doppler ultrasound showing no significant findings

The patient continued to have frequent visits to the ED for severe hypertension and recurrent hospitalizations for hypotension. Despite adding clonidine 0.1 mg twice daily, minoxidil 2.5 mg twice daily, chlorthalidone 12.5 mg daily, and carvedilol 25 mg twice daily, the patient continued to have spikes of BP with SBP as high as 200. The patient stated that his BP worsened overnight while supine but improved with ambulation. He reported severe anxiety associated with his labile hypertension and conveyed a frequent fear of sleeping overnight secondary to fears of developing a stroke. Renal artery Doppler ultrasound showed kidneys of normal size and echogenicity with known aortic aneurysm and no hydronephrosis, shadowing renal calculi, renal masses, perinephric fluid collections, or evidence of a hemodynamically significant renal arterial stenosis (Figure 3). He underwent a renal arteriogram, which showed <40% renal artery stenosis; vascular surgery deemed this degree of stenosis to be insignificant and felt this was not contributing to the patient’s labile hypertension. Cardiology was consulted, and losartan potassium 25 mg, half a tablet twice daily, was prescribed. Endocrinology was consulted for further evaluation. The patient underwent extensive laboratory workup, including urine cortisol, total metanephrines, renin, and serum aldosterone (Table 1), in addition to a complete blood count and comprehensive metabolic panel. The patient’s mildly elevated serum aldosterone was attributed to diuretic therapy. An ultrasound of the soft tissues of the head and neck was ordered to rule out paraganglioma and showed atherosclerotic peripheral vascular disease and no evidence of paraganglioma.

**Table 1:** Laboratory workup for suspected secondary hypertension

Name	Result	Reference Range
Cortisol free, urine	6.9 ug/L	–
Urine volume	2,700 mL	600-2000 mL
Cortisol-free, 24 hour	18.6 ug/day	<60.0 ug/day
Total metanephrines	629 ug/day	140-785 ug/day
Normetanephrine, urine	410 ug/day	88-444 ug/day
Metanephrines, urine	219 ug/day	52-341 ug/day
24-hour creatinine	1.4 gm/24h	1.0-2.0 gm/24h
Total catecholamines	56 ug/day	15-100 ug/day
Epinephrine, urine	5 ug/day	0-20 ug/day
Norepinephrine, urine	51 ug/day	15-80 ug/day
Dopamine, urine	192 ug/day	65-400 ug/day
Aldosterone, serum	29.9 ng/dL	≤39.2 ng/dL upright ≤23.2 ng/dL supine
Renin, direct	59.2 pg/mL	3.1-57.1 pg/mL

On subsequent follow-up four months later, the patient was continued on citalopram 20 mg daily with as-needed lorazepam 0.5 mg for management of his ongoing anxiety. His BP was controlled on carvedilol 25 mg twice daily, clonidine hydrochloric acid (HCl) 0.1 mg twice daily, and losartan 25 mg daily. The patient's BP has remained normotensive on this medication regimen.

## Discussion

From 2017 to 2020, hypertension was reported to be present in 115.3 million adults in the United States, of which only 43% were controlled with antihypertensives [6]. Uncontrolled hypertension is a major contributing factor in the progression of cardiovascular disease due to compensatory left ventricular wall thickening and eventual heart failure [7]. This emphasizes the importance of identifying the cause of elevated BP in order to reach a controlled state. When hypertension is refractory to current treatment guidelines, secondary causes are considered, such as resistant hypertension and pseudoresistance. Table 2 highlights the common causes of secondary hypertension as well as the workup and treatment for the different conditions.

**Table 2:** Commonly investigated causes of secondary hypertension GFR: glomerular filtration rate; US: duplex ultrasonography; CTA: computed tomography angiography; MRA: magnetic resonance angiography; ACE: angiotensin-converting enzyme; ARB: angiotensin receptor blocker; and ARR: aldosterone-to-renin ratio [8-12]

Causes of Secondary Hypertension	Signs and Symptoms	Laboratory Workup	Treatment
Renal parenchymal disease	Edema, polyuria, hematuria, anemia, weakness, fatigue	GFR <60 mL/min/1.73^2, urine albumin >30mg per 24 hours	Dietary sodium restriction, antihypertensives, diuretics
Renal artery stenosis	Asymptomatic to accelerated hypertension and renal insufficiency	>70% stenosis on duplex US, CTA, or MRA	ACE inhibitors/ARB therapy, endovascular stenting
Primary aldosteronism	Hypokalemia, fatigue, muscle weakness, muscle cramping	Morning ARR >30 or >20 with plasma aldosterone >16 ng/dL	Mineralocorticoid receptor antagonists, adrenalectomy
Pheochromocytoma	Paroxysmal headaches, diaphoresis, tachycardia	24-hour urine fractionated metanephrines and catecholamines	Adrenalectomy
Cushing syndrome	Glucose intolerance, acne, osteoporosis, obesity, moon facies, muscle wasting	24-hour urine cortisol or low-dose dexamethasone suppression	Surgical tumor resection, medication, radiation therapy, adrenalectomy

Our case presents an elderly male patient with a 60-pack-year smoking history, previous EVAR after AAA dissection, and no prior hypertension who began suddenly experiencing uncontrollable high BP. Pseudoresistance is the least invasive method to initially evaluate by thoroughly examining patient adherence to treatment, BP measurement technique, and the white coat effect [13]. In our patient, pseudoresistance was eliminated as medication adherence and BPs were consistently managed and maintained. Daytime and nighttime BPs, as well as medication timing and dosages, were thoroughly recorded and reported at each follow-up. This information became pertinent to identifying any minor medication adjustments or changes resulting in significant hypotensive episodes.

The patient was presumed to have primary hypertension, but despite management with four different antihypertensive medications, our patient continued to have BP spikes upwards of 200 mmHg systolic, which then met the criteria to be considered treatment-resistant. With the exception of a slightly elevated renin level (Table 1), all of our patient’s laboratory values returned within normal limits. This excluded primary hyperaldosteronism, Cushing syndrome, and pheochromocytoma as possible diagnoses. Further imaging studies with renal ultrasound and head and neck soft tissue ultrasound eliminated the possibility of renal artery stenosis and paraganglioma, respectively.

With no explanation for our patient’s resistant hypertension, we began investigating a potential cause in relation to his history of ruptured AAA and EVAR. The complication rate following EVAR is between 16% and 30%, with the most common being endograft-related complications, limb kinking or occlusion, ischemia, embolic strokes, and post-implantation syndrome [14]. However, our patient’s symptoms, laboratory values, and imaging studies did not correlate with these complications.

Gal-Oz et al. report a case of malignant hypertension two months following an EVAR of suprarenal and juxtarenal abdominal aneurysms in a 64-year-old male. Renal duplex ultrasound revealed near-complete occlusion of the left renal artery due to thrombosis caused by perioperative cessation of antiplatelet therapy [15].

Daoud et al. similarly reported a case of uncontrollable hypertension two months after an aortic dissection repair in an 83-year-old female. After hospitalization for hypertensive encephalopathy, labile BP and CTA findings of decreased blood flow to the right kidney suggested renovascular hypertension due to damage to the aortic baroreceptors during surgery. Labetalol and losartan, as shown in the current case, were successful in controlling the inappropriate levels of renin-angiotensin-aldosterone system activation without hypotensive episodes [16].

AAAs are highly dangerous due to the mortality rate of their complications. Patients who survive a dissection and repair remain at high risk and need strict surveillance to detect the onset of any disturbances. Controlling BP is an important aspect of maintaining their care, and beta-blockers have been shown to play a role in this control as well as decreasing the need for reoperation [17-18].

Upon searching the literature, we found that reports of resistant hypertension following aortic dissection were very limited. Our case demonstrates an example of treatment-resistant hypertension without an identifiable cause in a patient with a history of aortic dissection and EVAR. This supports the need for close follow-up and evaluation and the possible necessity for individualized antihypertensive therapy to reach optimal BPs and improve their long-term survival.

## Conclusions

Hypertension is a common but dangerous pathology with an often unknown origin. Underlying medical disease and other variables can play a role in the development of secondary hypertension. In our case, labile hypertension developed following an EVAR on an AAA. It is our hope that our case can add to the growing body of knowledge surrounding associations, experiences, and factors that can influence the development of labile, treatment-resistant hypertension. In our case, we were able to successfully control the patient's BP with carvedilol, clonidine HCl, and losartan.
